# Supplementary material for: Mitochondrial gene expression in single cells shape pancreatic beta cells' sub-populations and explain variation in insulin pathway
Source: Sci Rep. 2021 Jan 11;11:466. doi: 10.1038/s41598-020-80334-w (PMC7801437; doi:10.1038/s41598-020-80334-w)
Supplement: Supplementary file 1 — Supplementary Figures. [file 41598_2020_80334_MOESM1_ESM.docx]

**Mitochondrial gene expression in single cells shape pancreatic beta cells' sub-populations and explain variation in insulin pathway**

Medini, H.^1^, Cohen, T.^1^ and Mishmar, D.^1*^

1 Department of life Sciences, Ben-Gurion University of the Negev, Beer Sheva 8410501, Israel

*Corresponding author:

Dan Mishmar, PhD

Department of life Sciences

Ben-Gurion University of the Negev

Beer Sheva Israel 8410501

Email: [dmishmar@bgu.ac.il](mailto:dmishmar@bgu.ac.il)

Supplementary Figures S1-S22:


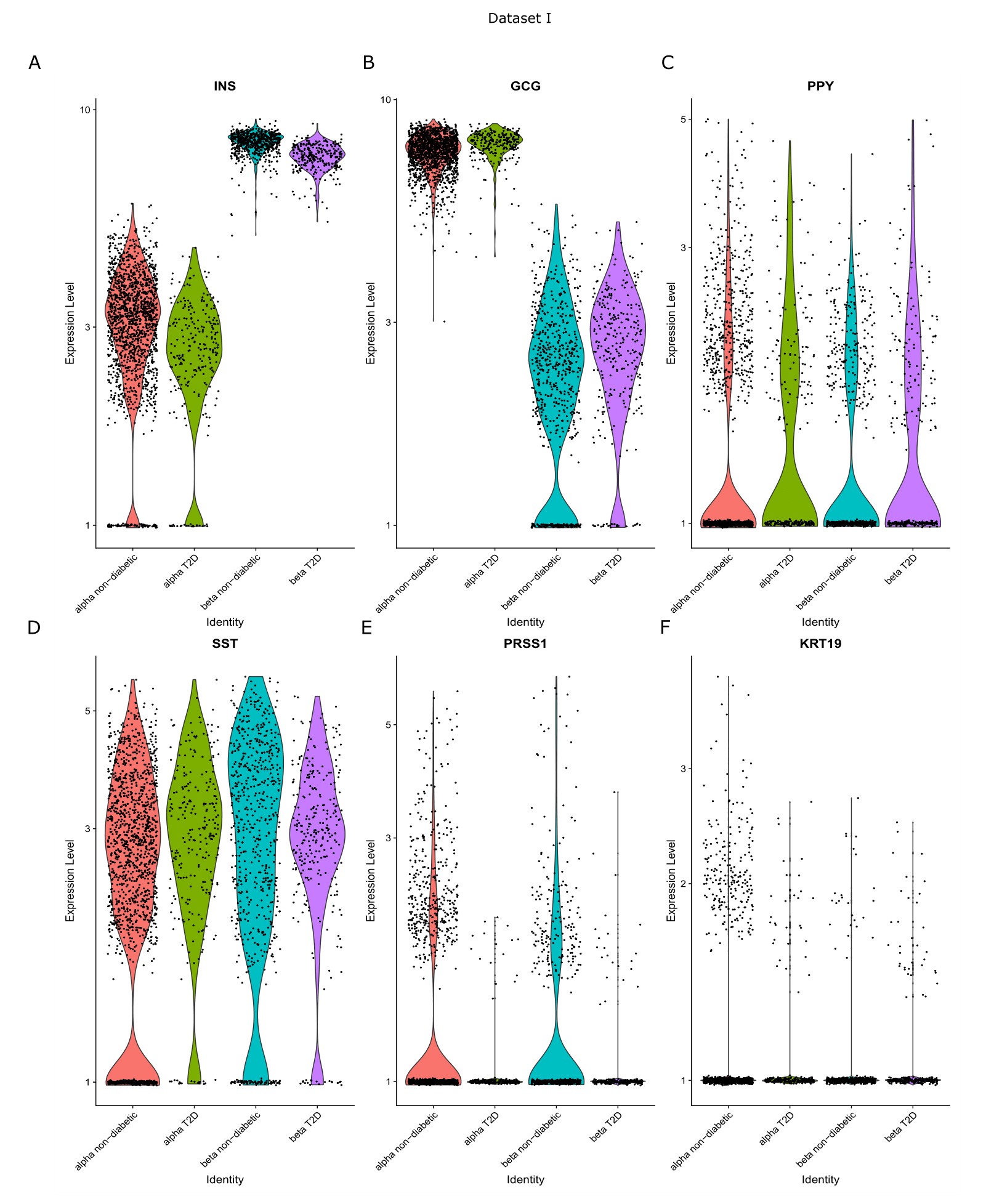


**Figure S1 - Violin distribution plots of selected marker genes' expression in pancreatic alpha and beta cells, in Dataset I.** Notice that alpha and beta cells display strong expression of their markers (i.e., Ins and GCG, respectively). Expression of (A) INS (a marker of pancreatic beta cells), (B) GCG (a marker of alpha cells), (C) PPY (a marker of gamma cells), (D) SST (a marker of delta cells), (E) PRSS1 (a marker of acinar cells), (F) KRT19 (a marker of ductal cells).


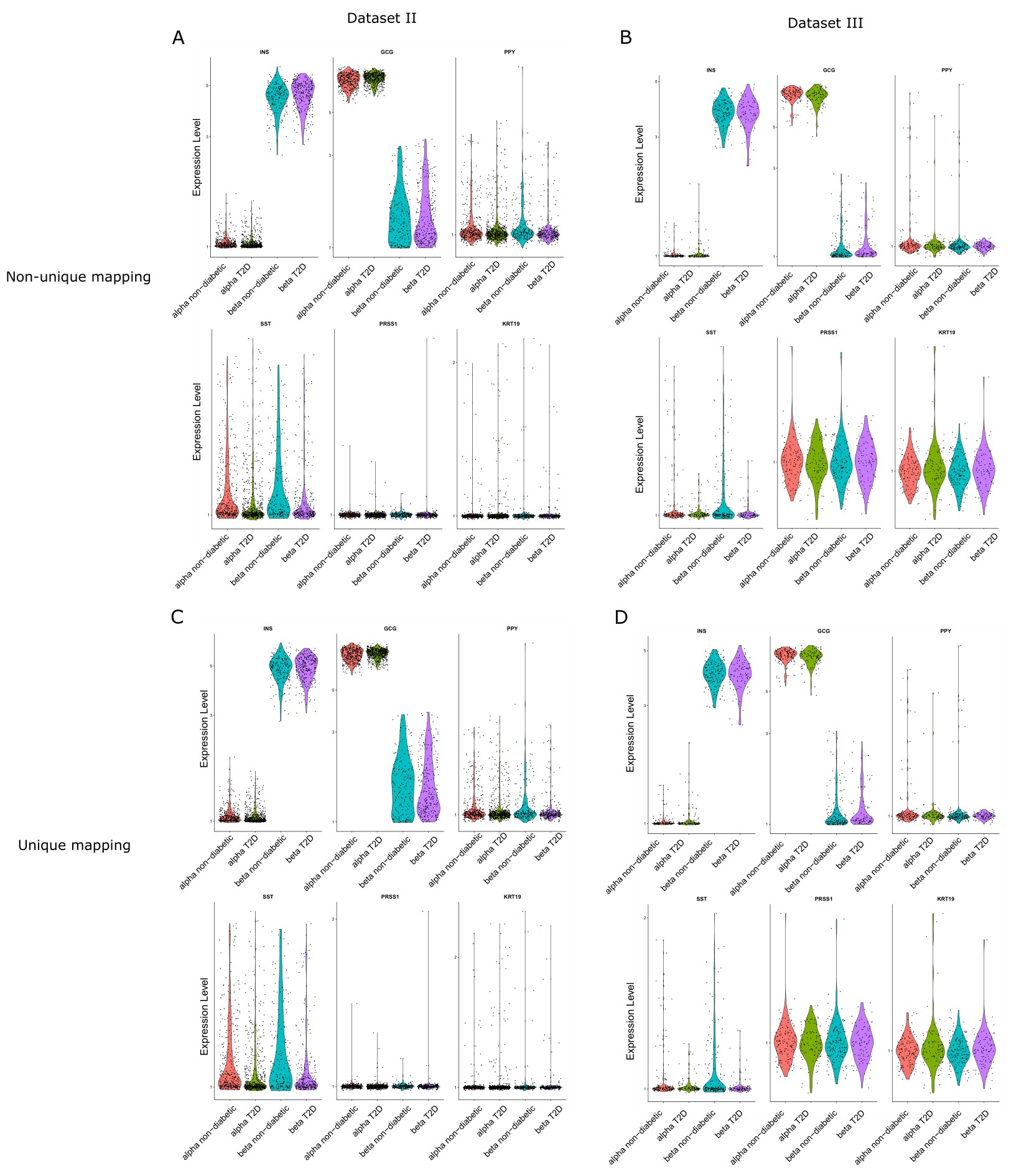


**Figure S2 - Violin distribution plots of selected marker genes' expression in pancreatic alpha and beta cells, in Datasets II and III.** Notice that alpha and beta cells display strong expression of their markers (i.e., Ins and GCG, respectively). Shown are the expression levels of INS (a marker of beta cells), GCG- a marker of alpha cells, PPY- a marker of gamma cells, SST- a marker of delta cells, PRSS1- a marker of acinar cells, KRT19- a marker of ductal cells. (A) Datasets II (Nonunique mapping), (B) Dataset III (Nonunique mapping), (C) Datasets II (Unique mapping), (D) Dataset III (Unique mapping).


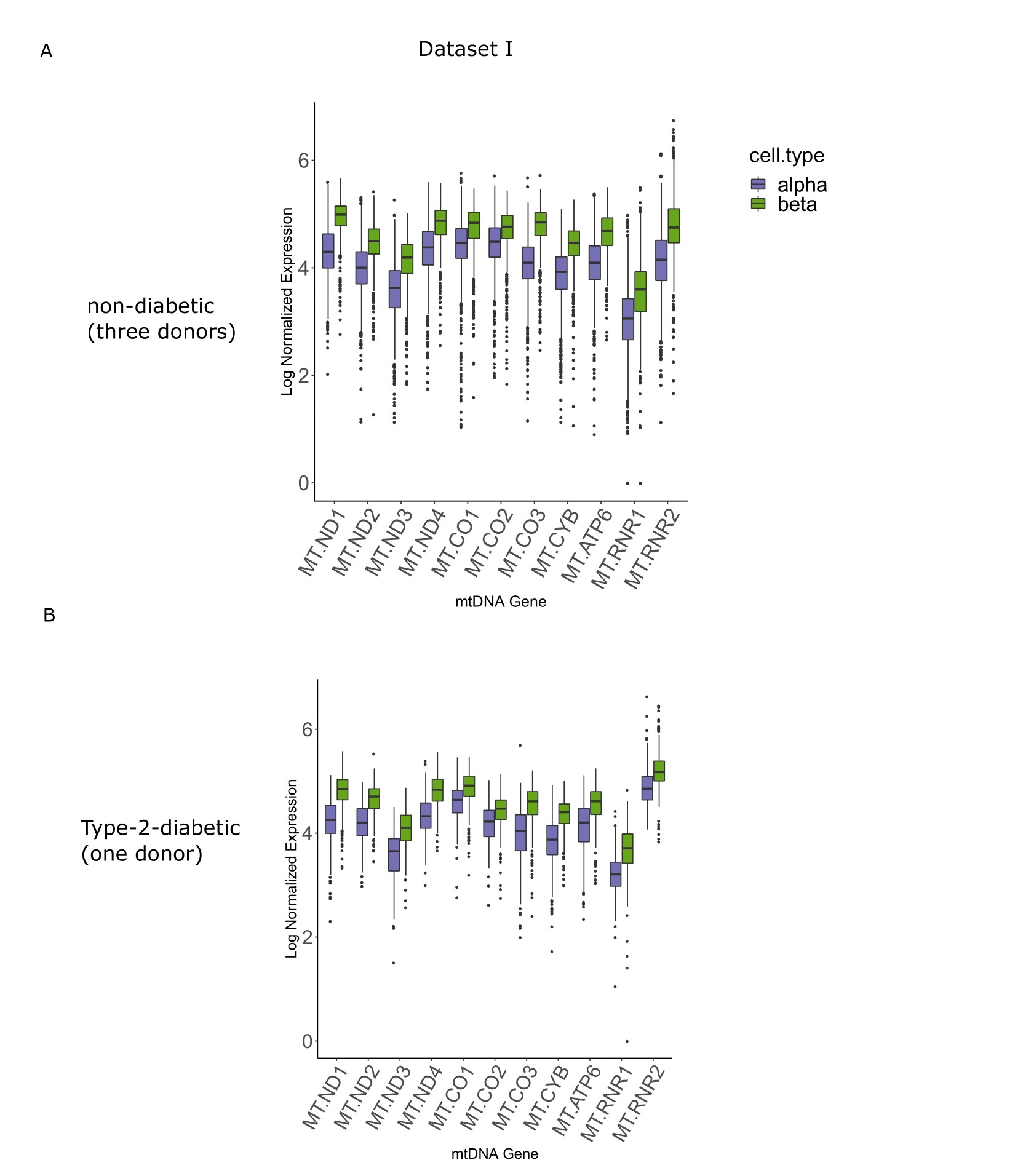


**Figure S3** **- Beta cells display higher mtDNA gene expression regardless of diabetes status in Dataset I.** Box plot of the mtDNA gene expression in the total collection of alpha and beta cells from (A) nondiabetic (ND) donors and (B) T2DM donor (Dataset I). Statistical results and p-values are displayed in Table S2.


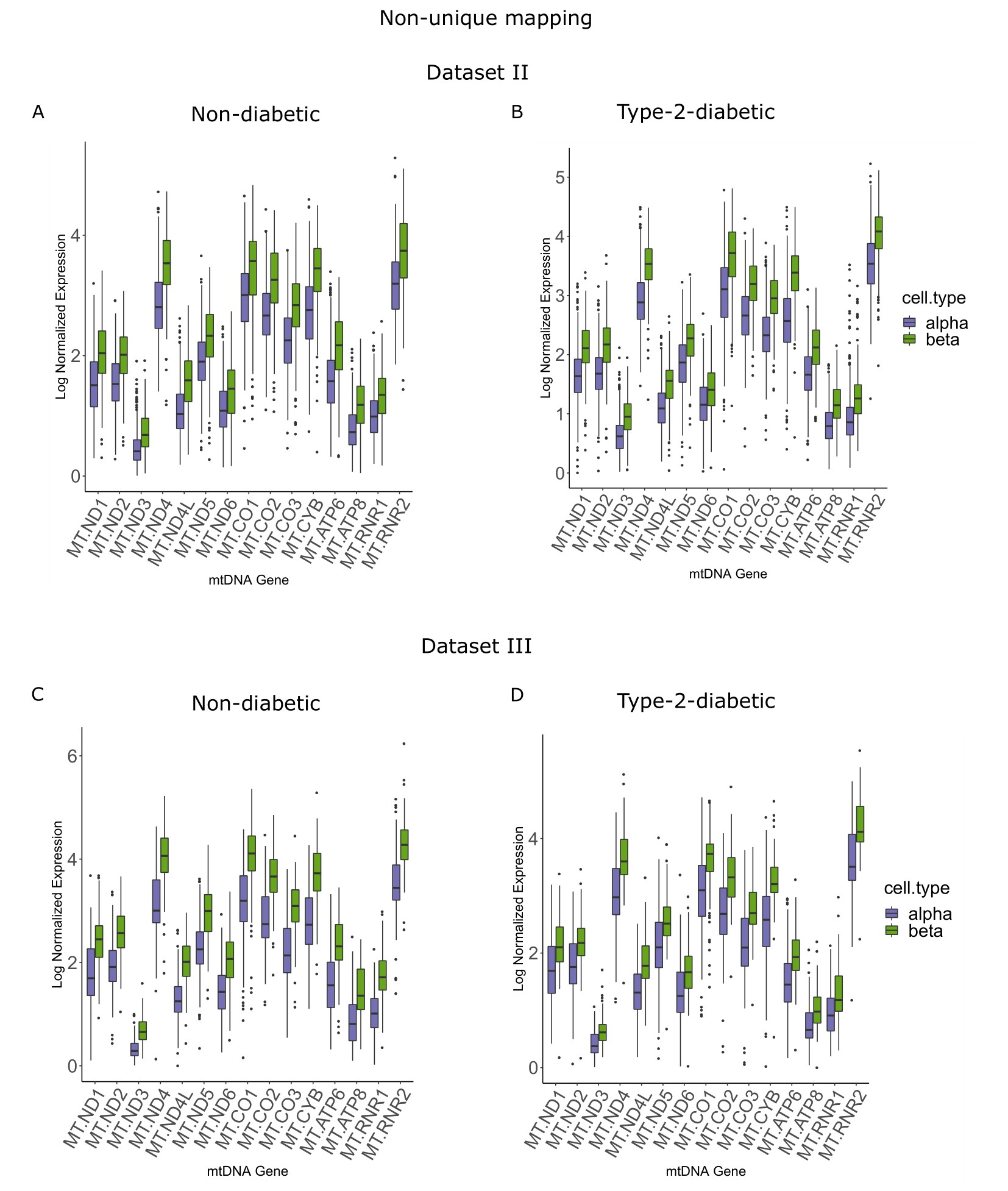


**Figure S4** **- Beta cells have higher mtDNA gene expression regardless of diabetes status (non-unique mapping) in Datasets II and III.** Box plot of the mtDNA gene expression pattern of the total collection of alpha and beta cells from Dataset II and III: Dataset II- (A) ND donors and (B) T2DM donors. Dataset III- (C) ND donors and (D) T2DM donors. Statistic results and p-values are displayed in Table S2.


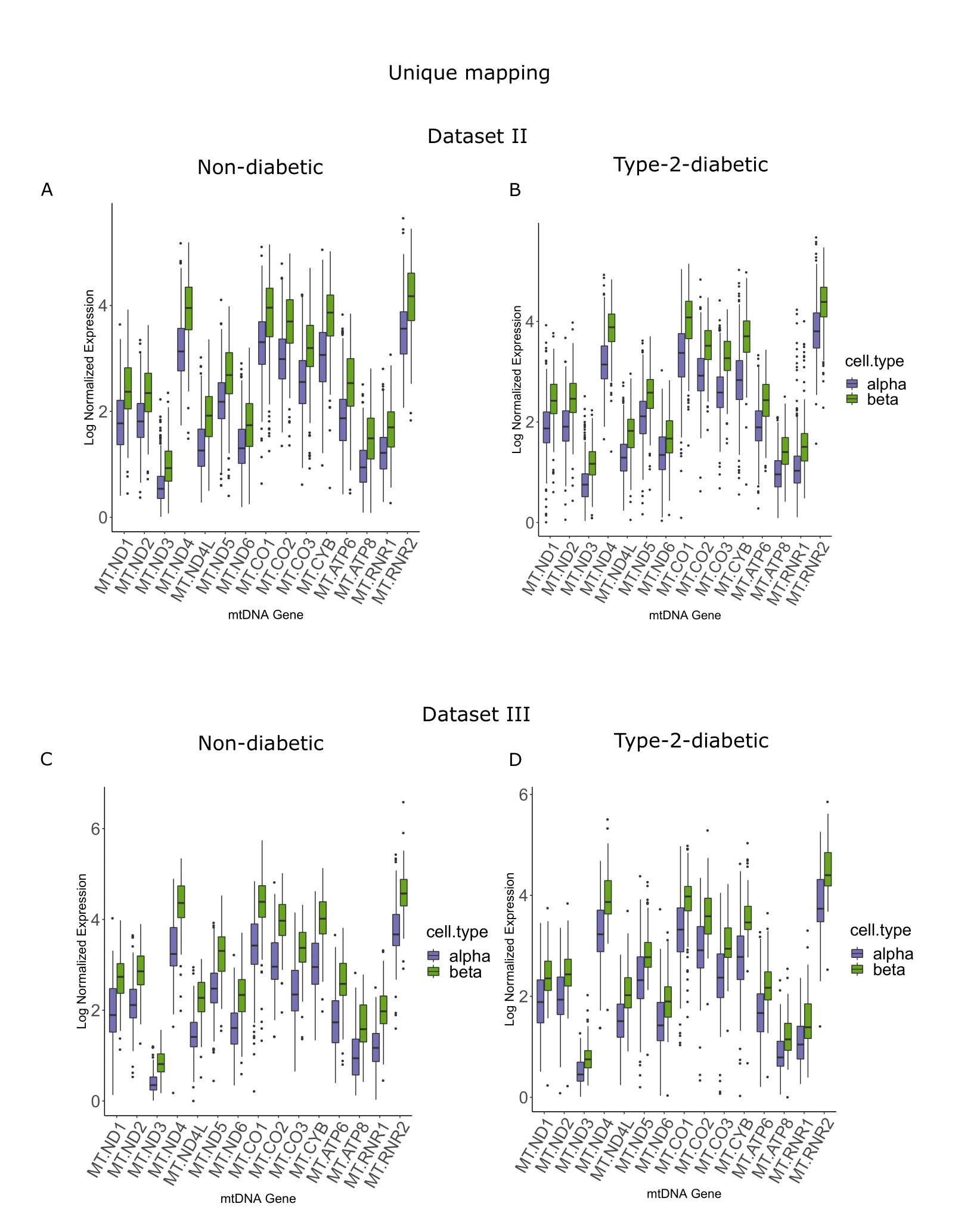


**Figure S5** **- Beta cells have higher mtDNA gene expression regardless of diabetes status (unique mapping) in Datasets II and III.** Box plot of the mtDNA gene expression pattern of the total collection of alpha and beta cells from Dataset II and III: Dataset II- (A) ND donors and (B) T2DM donors. Dataset III- (C) ND donors and (D) T2DM donors. Statistic results and pvalues are displayed in Table S2.


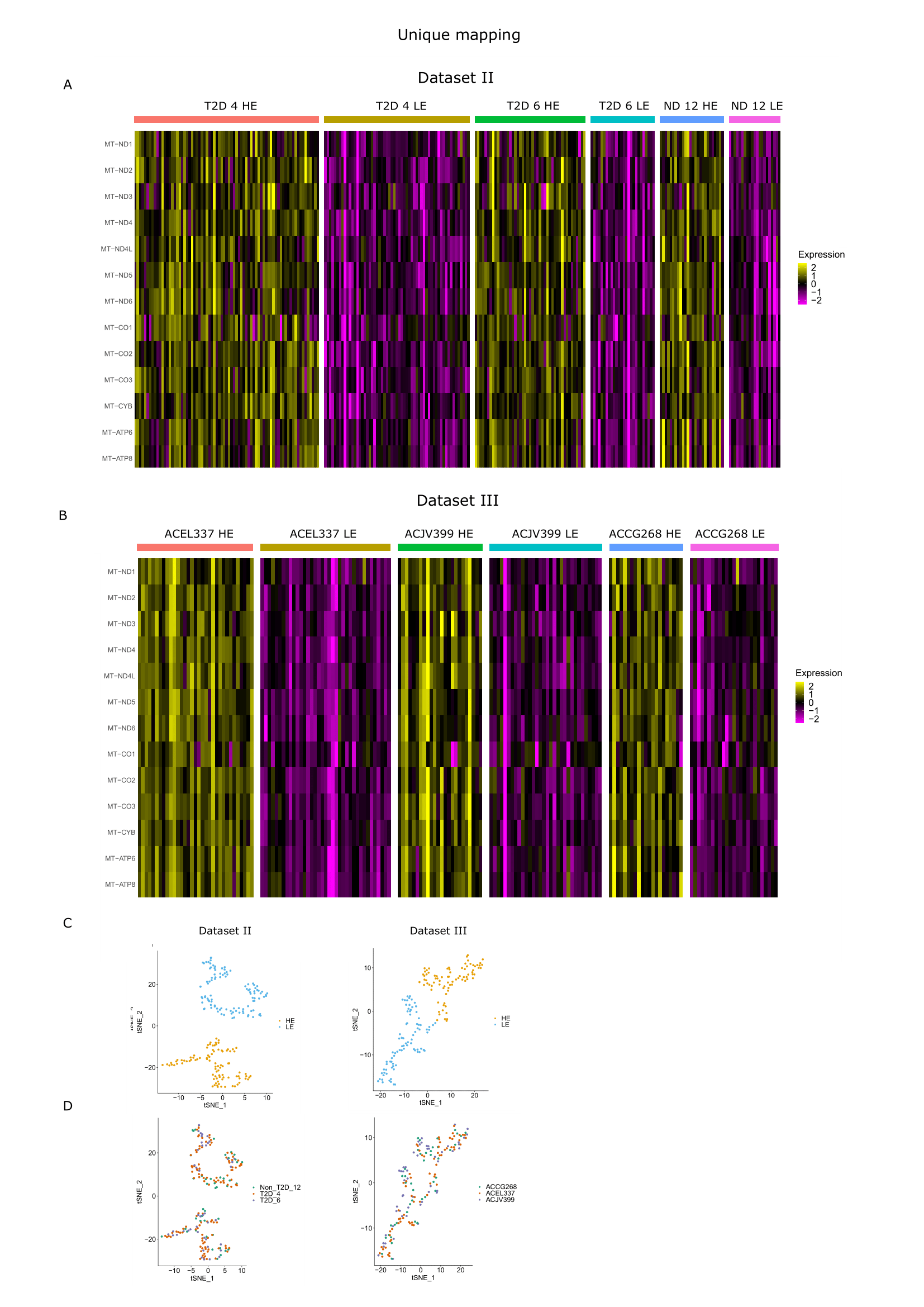


**Figure S6** **– Beta cells from Datasets II and III (unique mapping) diverged according to mtDNA gene expression into high (HE) and low (LE) subgroups.** Heatmaps of (A) beta cells from Dataset II (B) beta cells from Dataset III clustered by mtDNA gene expression (purple- low expression, yellow- high expression). tSNE of beta cells from datasets II and III clustered by mtDNA gene expression colored by (C) subgroup identity, (D) donor identity.


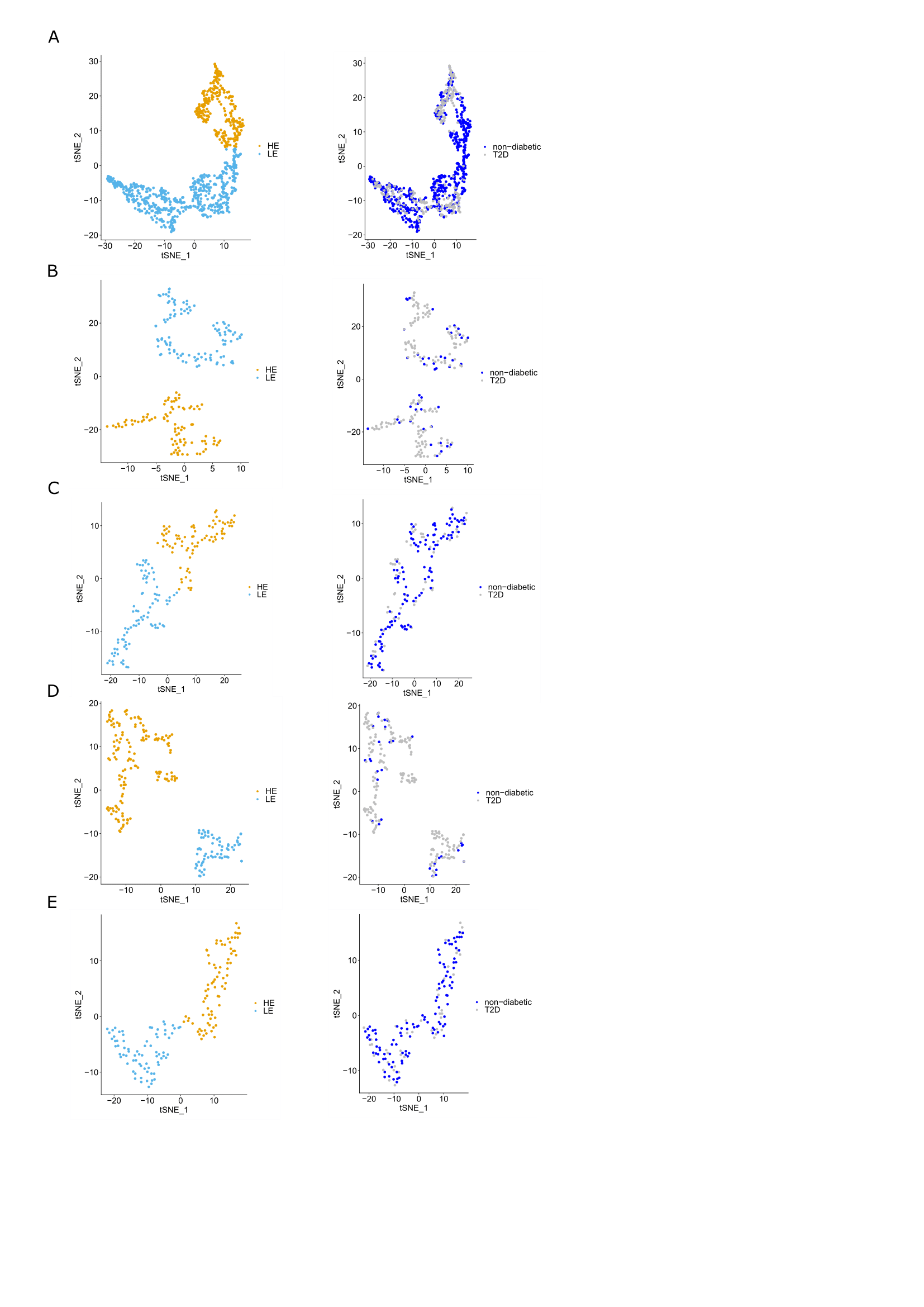


**Figure S7** **– Beta cells subgroups contain cells from both healthy and diabetic human individuals.** (A) tSNE of beta cells distribution in human Datasets I. (B-C) tSNE of betac cells distribution in human Datasets II and III (unique mapping). (D-E) tSNE of beta cells distribution in Datasets II and III (non-unique mapping). Blue: non-diabetic donors, grey: type-2-diabetic (T2D patients).


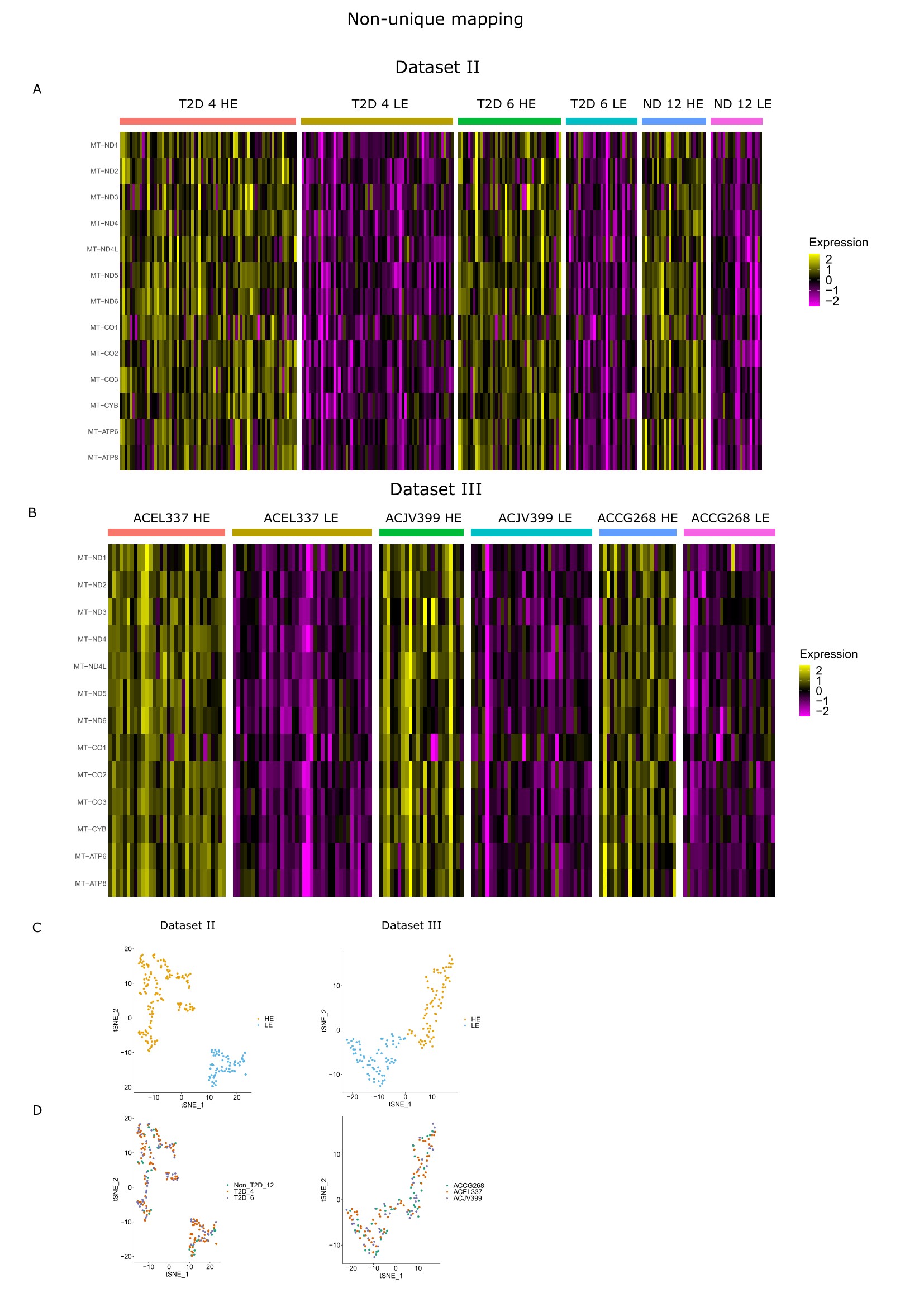


**Figure S8** **– Beta cells from Datasets II and III (non-unique mapping) diverged according to mtDNA gene expression into high (HE) and low (LE) subgroups.** Heatmaps of (A) beta cells from Dataset II (B) beta cells from Dataset III clustered by mtDNA gene expression (purple- low expression, yellow- high expression). tSNE of beta cells from Datasets II and III clustered by mtDNA gene expression colored by (C) subgroup identity, (D) donor identity.


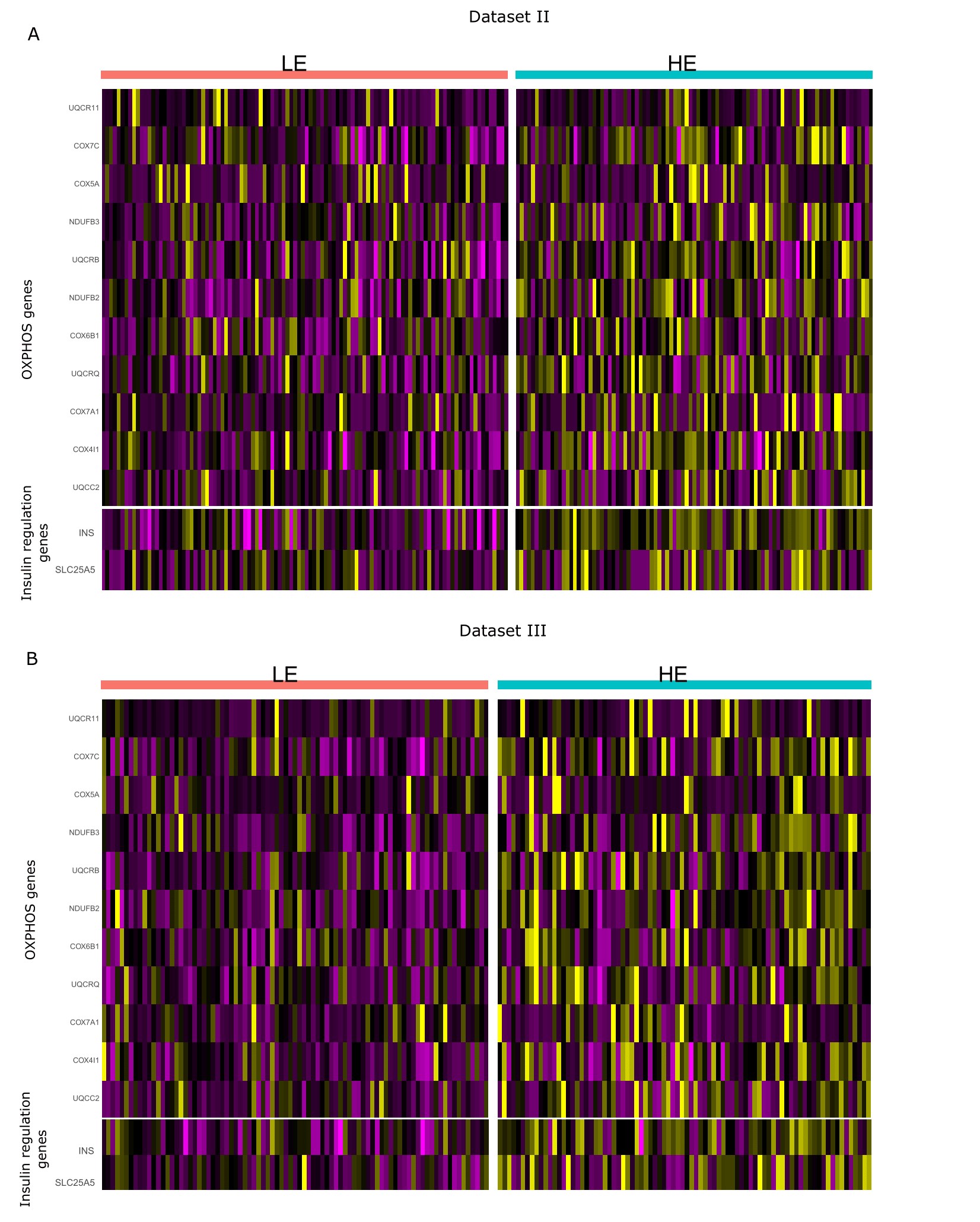


**Figure S9** **– Heatmaps showing differential nuclear DNA-encoded gene expression between the LE and HE beta cells subgroups.** Shown are the results from (A) Datasets II and (B) Dataset III, after unique mapping. In both A and B, the upper panel corresponds to OXPHOS genes, and lower panel corresponds to Insulin regulatory genes. Purple- low expression, yellow- high expression. Statistical results are summarized in Table S3.


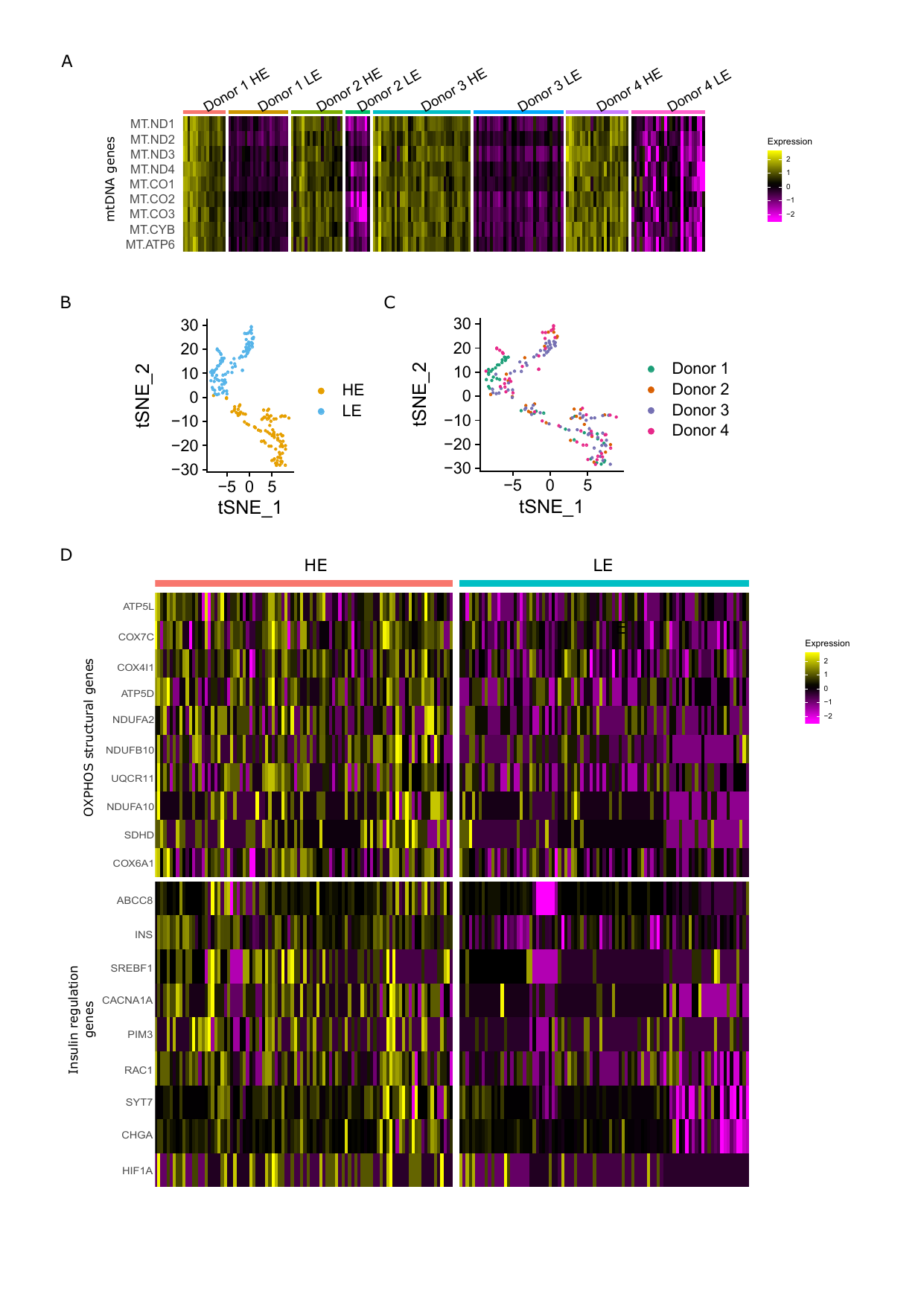


**Figure S10 - Human mtDNA gene expression analysis revealed two distinct beta cell clusters with either high (HE) or low (LE) mtDNA gene expression using a threshold of 10% mtDNA reads from Dataset I (N=185 beta cells)**. (A) Heatmap showing the subgroups of cells with either high (HE) or low (LE) mtDNA gene expression, per tested individual (donor); (B-C) tSNE distribution of beta cells from the four donors (Dataset I) showing two subgroups of beta cells with high and low mtDNA gene expression (HE and LE, respectively). Donor identity are colour coded as indicated. (D) Heatmap showing the significant differentially expressed genes per cell cluster, per individual (after FDR correction). Upper panel -OXPHOS structural genes; lower panel- genes involved in insulin regulation. Purple- low expression, yellow- high expression.


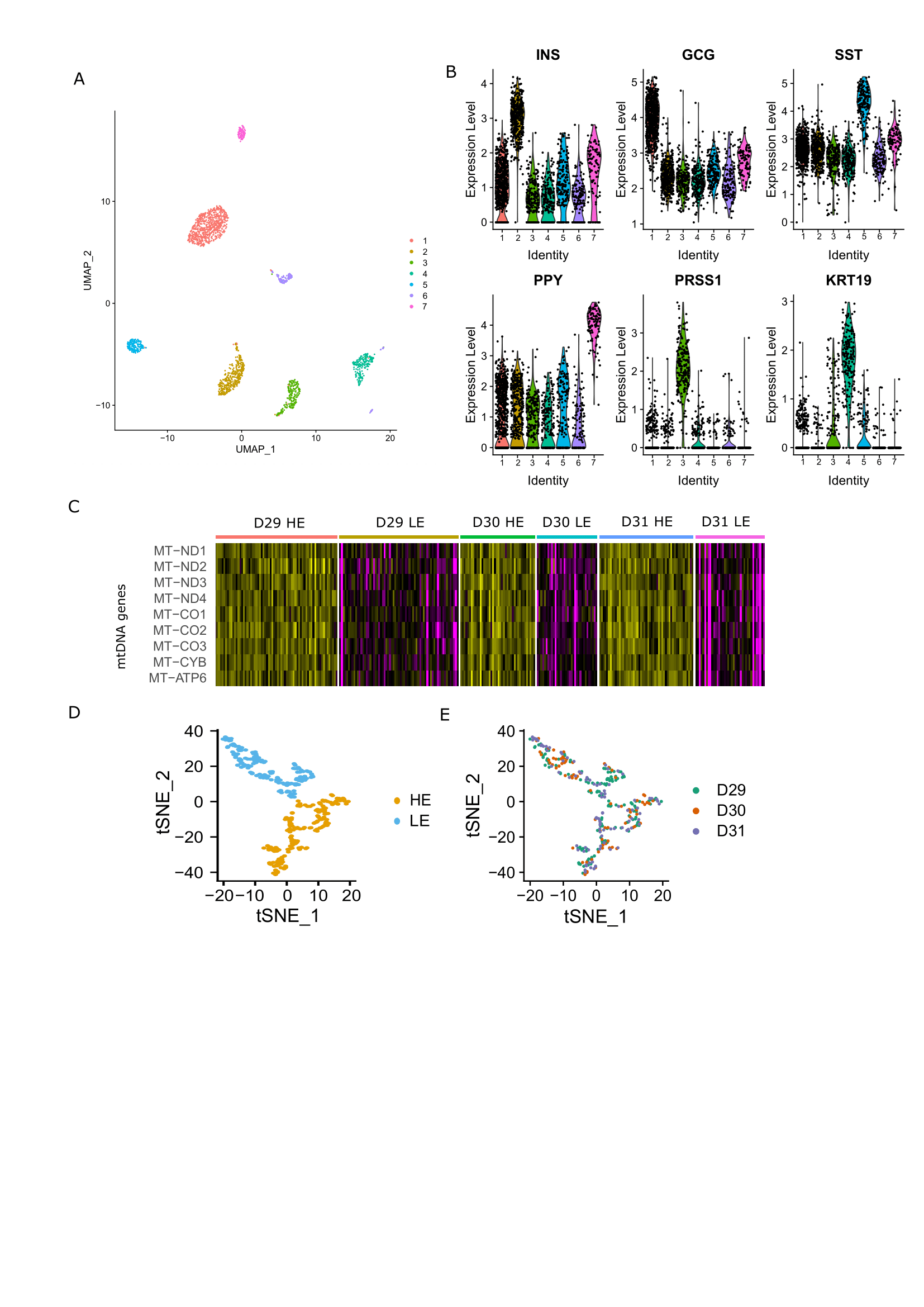


**Figure S11** **– Human mtDNA gene expression analysis of Dataset IV revealed the two distinct beta cell clusters with either high (HE) or low (LE) mtDNA gene expression.** (A) UMAP distribution enabled using the top 2000 variable genes (defined by Seurat) to identify six cell clusters (notice the colour code). (B) Violin distribution plots of marker genes' expression in pancreatic cells. Notice that INS expression (a marker of pancreatic beta cells) appears in cluster 2 (see A), GCG (a marker of alpha cells) is a marker of cells cluster 1 (see A), SST (marker gene of delta cells-cluster 5), PPY (marker genes of gamma cells –cluster 7), PRSS1 (a marker of acinar cells – cluster 3), KRT19 (a marker of ductal cells- cluster 4) and cells cluster 6 which was not defined by either of the mentioned marker genes. (C) Heatmap of beta cells clustered by mtDNA gene expression per tested individual. tSNE of beta cells clustered by mtDNA gene expression and coloured by either (D) subgroup identity (i.e. LE and HE), or according to (E) samples identity – D29-D30.


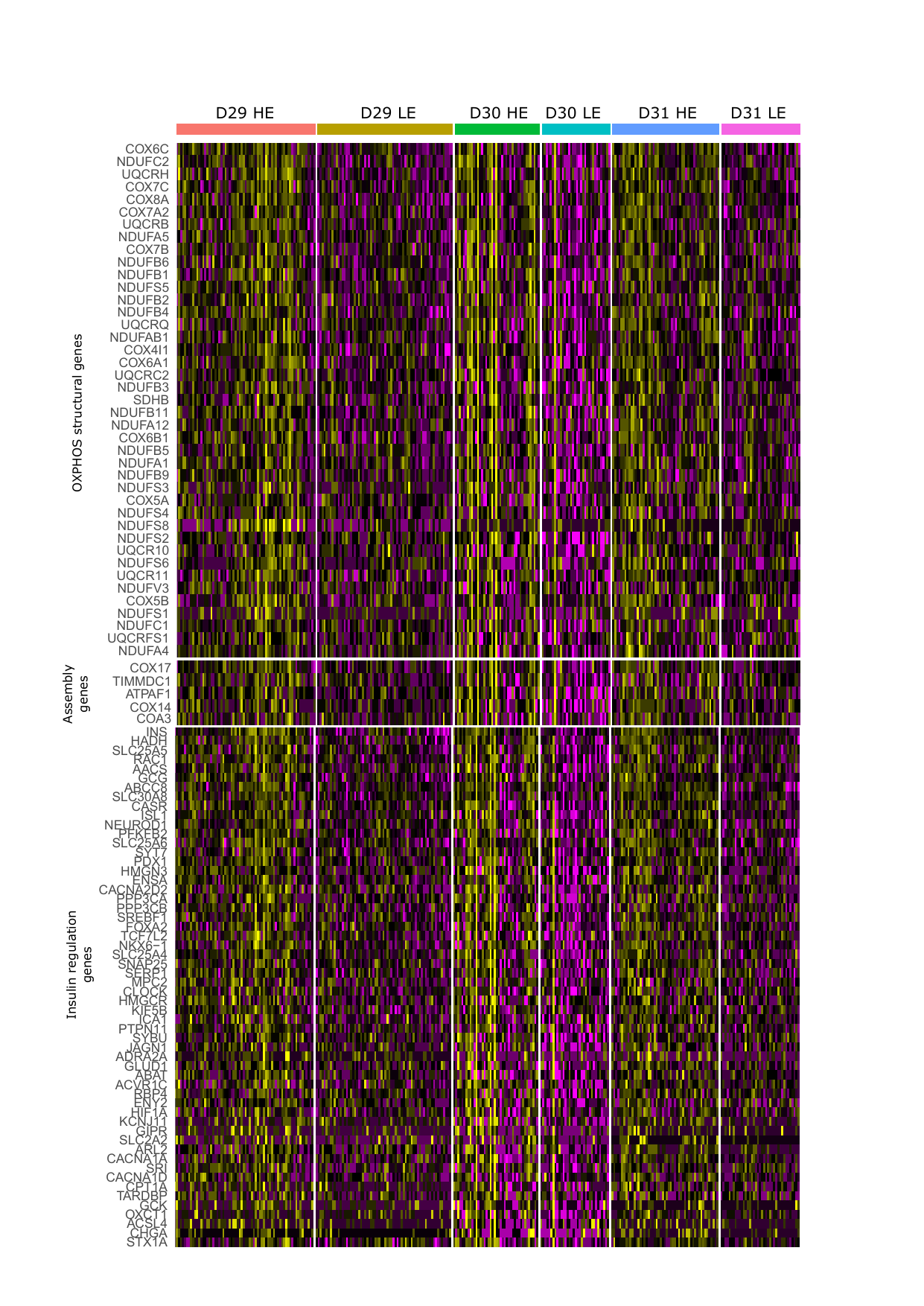


**Figure S12** **– Heatmap demonstration of nuclear DNA-encoded gene expression differences between the HE and LE cells' sub-clusters in Dataset IV.** Shown are the results of significant differentially expressed genes (negbinom test) per cell cluster (after FDR correction). Purple- low expression, yellow- high expression. The corresponding statistical results are summarized in Table S3.


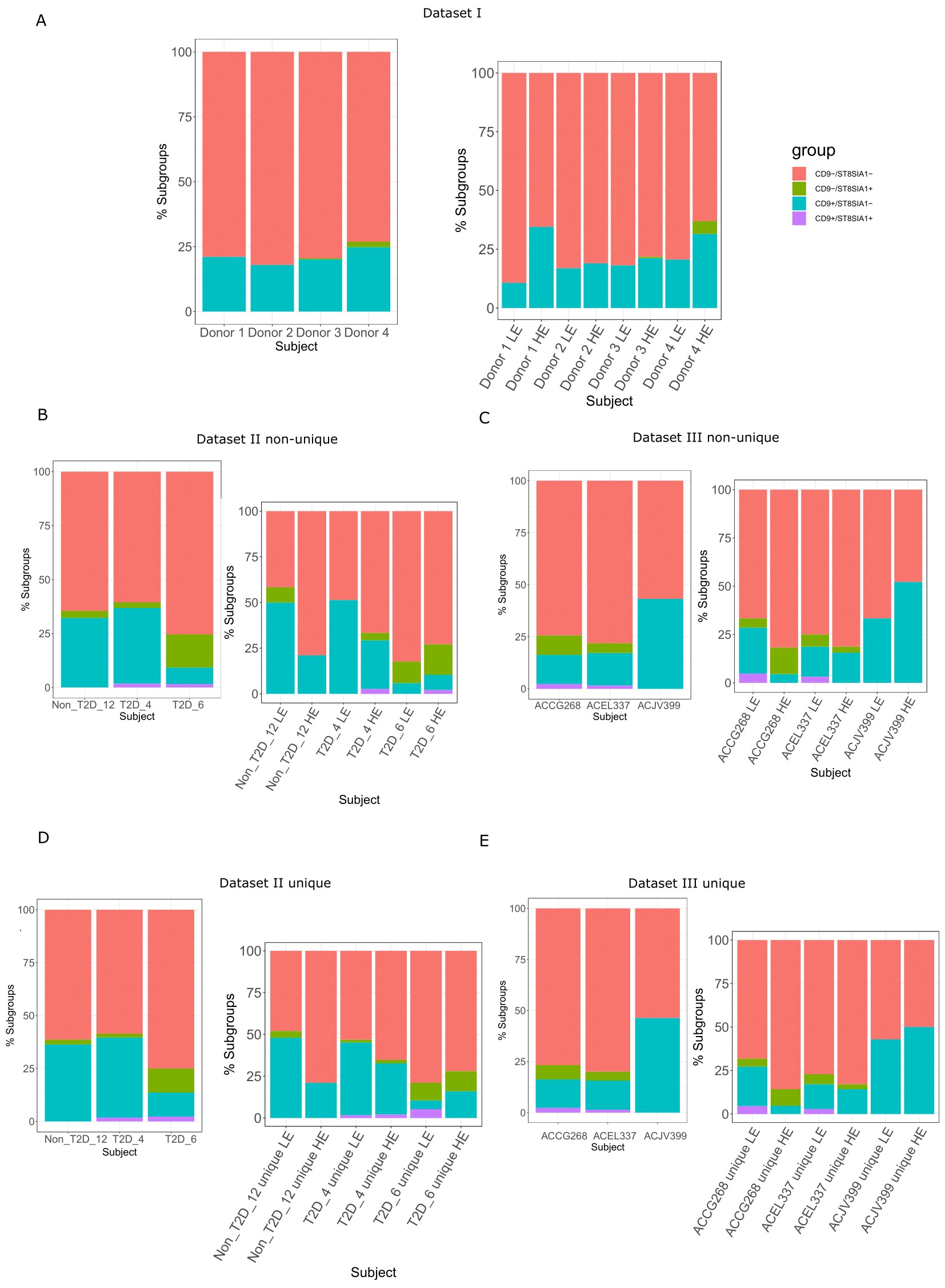


**Figure S13** **- Plots showing lack of association between the expression of nuclear antigens CD9 and ST8SIA1 in the LE and HE cellular subgroups.** (A) Dataset I, (B) Dataset II nonunique mapping, (C) Dataset III non-unique mapping, (D) Dataset II unique mapping, (E) Dataset III unique mapping.


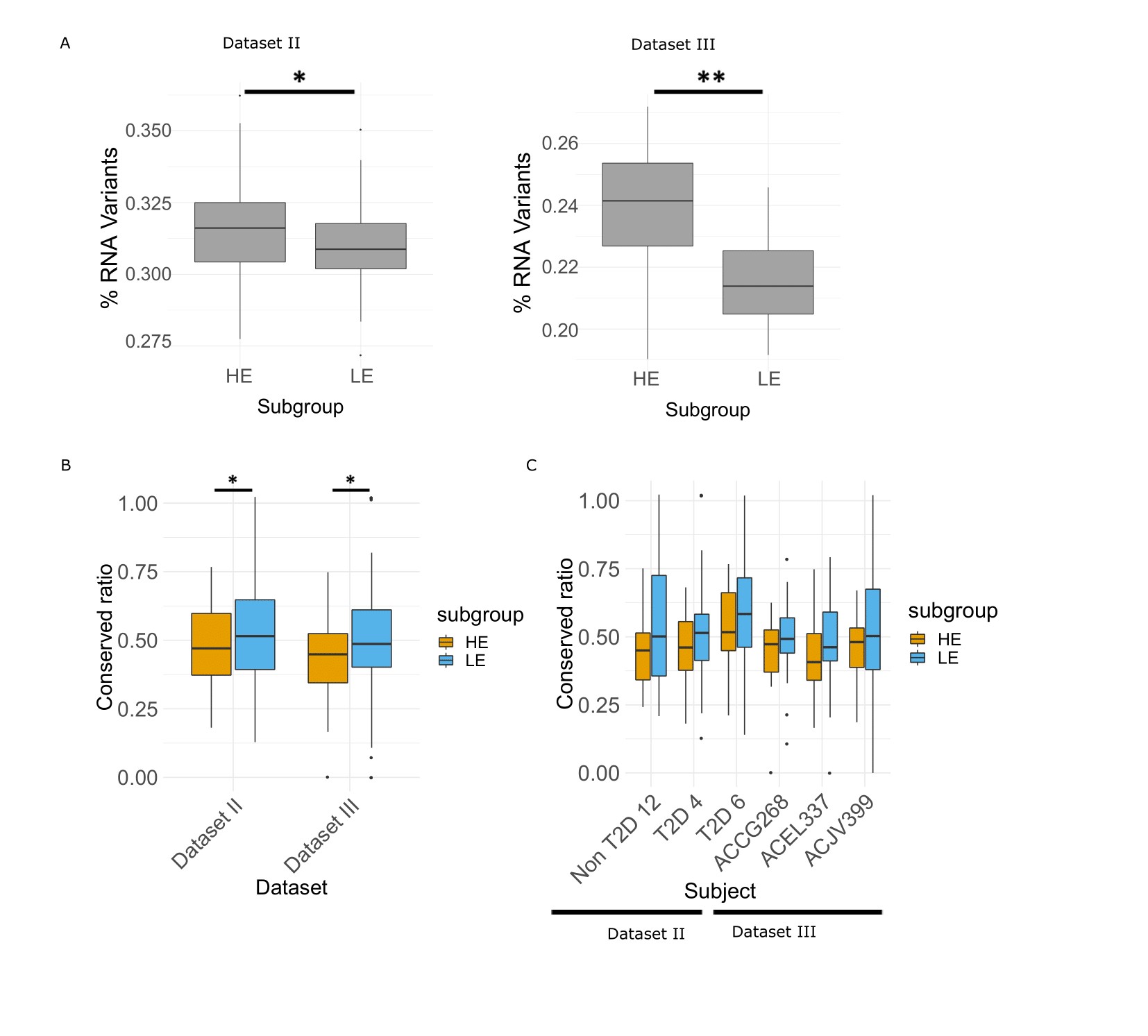


**Figure S14 - Human mt-RNA mutation patterns display a tendency towards higher mutational repertoire and lower conservation score (see Methods) in the HE subgroup in each of the tested six individuals.** Box plots reflect the distribution of calculated conserved ratios of LE and HE groups from the tested individuals of human Datasets II and III.


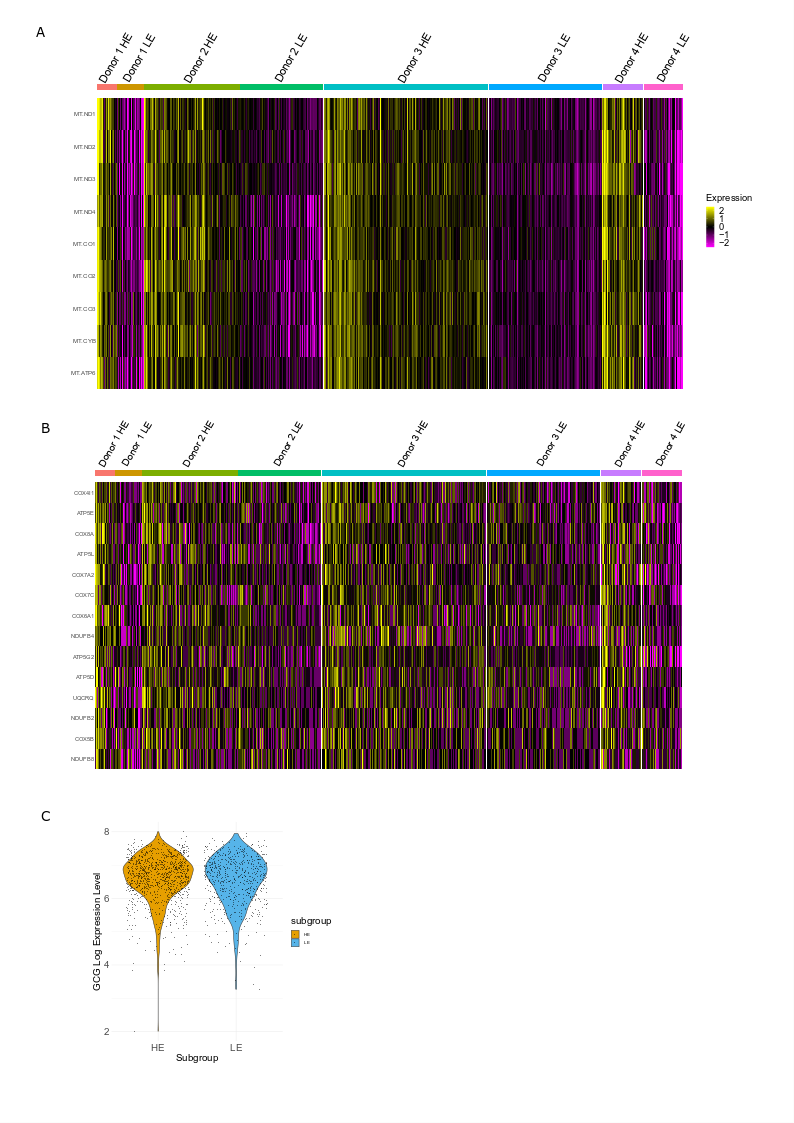


**Figure S15 - Pancreatic alpha cells are divided into two sub-groups according to mtDNA gene expression.** (A) Heatmap of significant differentially expressed genes per cluster per individual (after FDR correction) of mtDNA-encoded transcripts, (B) OXPHOS genes (purple- low expression, yellow- high expression). High and low mtDNA gene expression cell groups are indicated as HE and LE, respectively. (C) Violin plots showing the distribution of GCG gene expression per subgroup. Shown are the results from Dataset I.


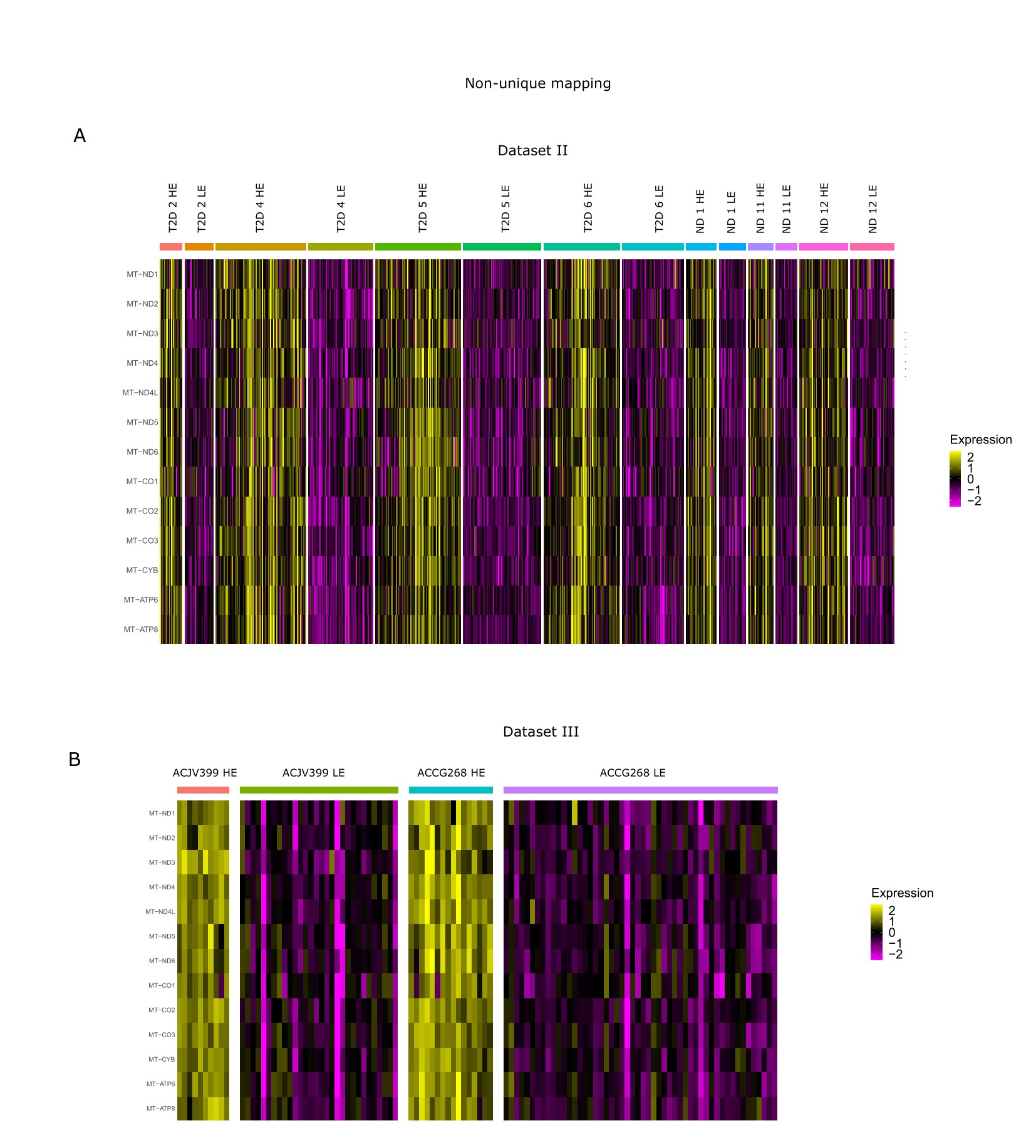


**Figure S16** **– Human pancreatic alpha cells are divided into two sub-groups according to mtDNA gene expression (non-unique mapping, Datasets II, III).** Heatmaps of mtDNA genes per cluster per individual (purple- low expression, yellow- high expression). High and low mtDNA gene expression cellular subgroups are HE and LE, respectively. (A) Dataset II, (B) Dataset III.


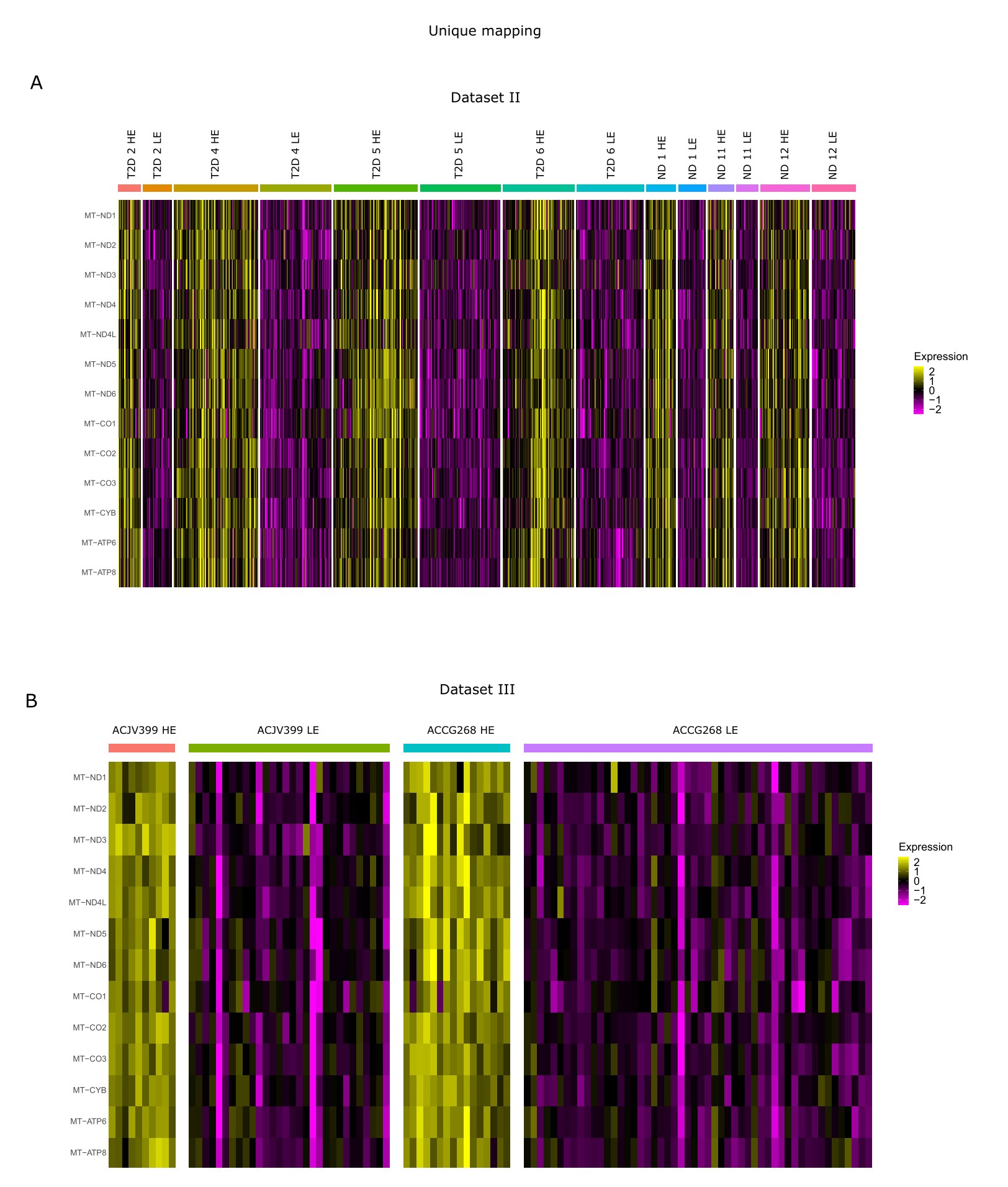


**Figure S17** **– Human pancreatic alpha cells are divided into two sub-groups according to mtDNA gene expression (unique mapping, Datasets II, III).** Heatmaps of mtDNA genes per cluster per individual (purple- low expression, yellow- high expression). (A) Dataset II, (B) Dataset III.


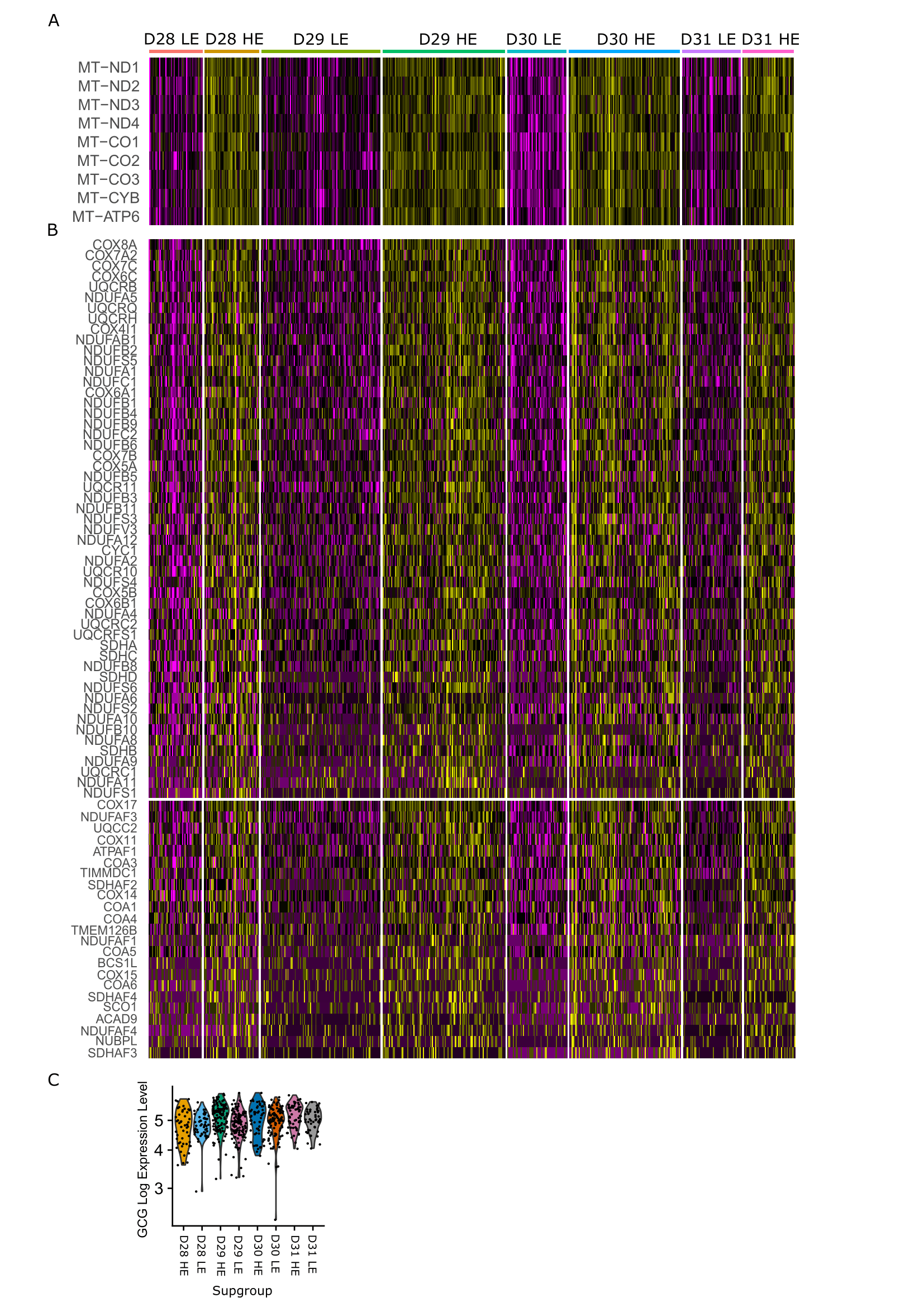


**Figure S18** **– Heatmaps showing differential mitochondrial gene expression between the LE and HE alpha cells subgroups in dataset IV.** (A) Heatmap of mtDNA genes per cluster per individual. (B) Heatmap of significant differentially expressed nDNA OXPHOS genes- upper panel- structural OXPHOS genes, lower panel- assembly genes (purple- low expression, yellow- high expression). (C) GCG gene expression per cluster per dataset, High and low mtDNA gene expression sign as HE and LE, respectively. Only two donors out of four, D28 and D30, had significantly higher GCG gene expression in the HE subgroup (p<1x10^-4^).


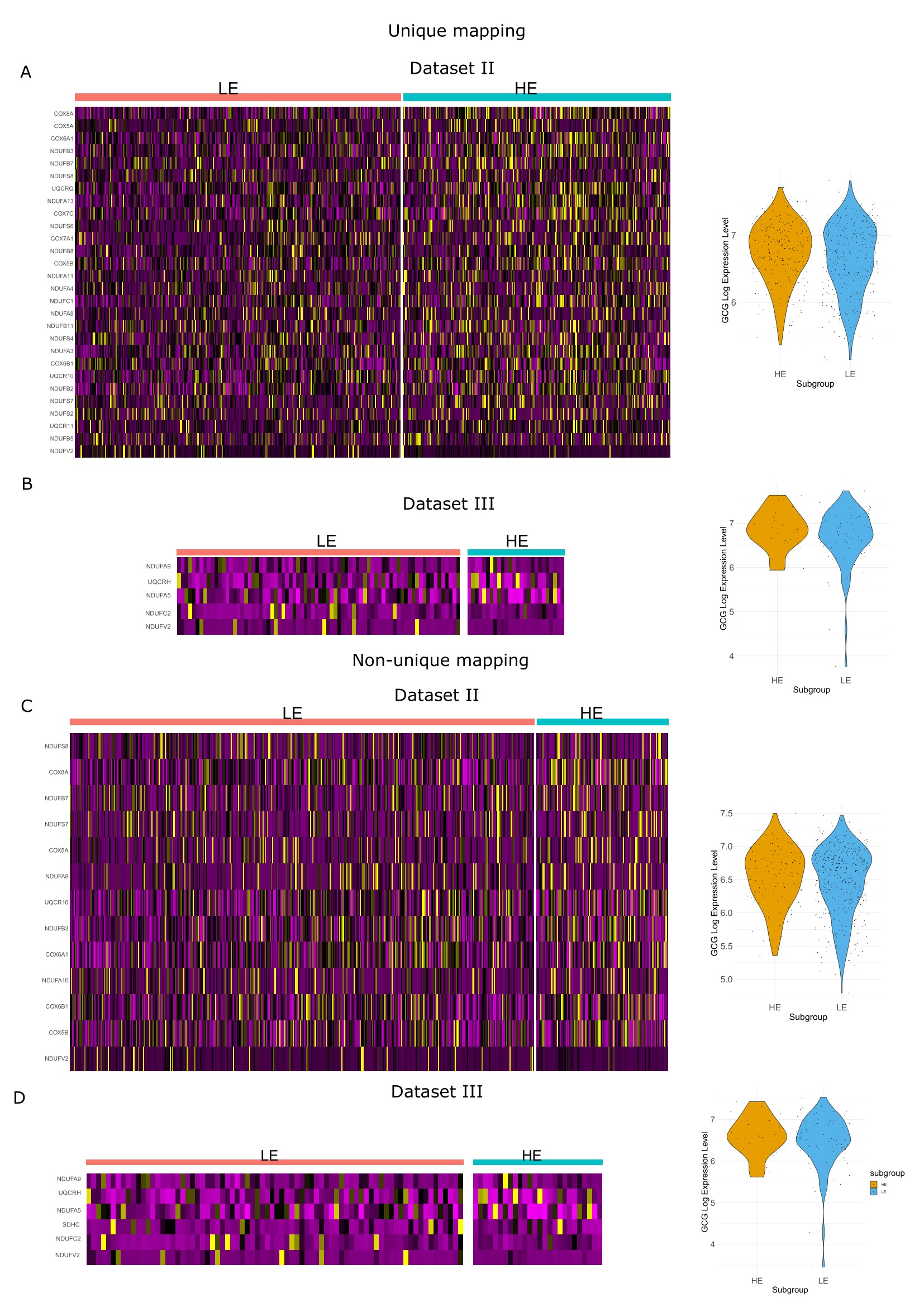


**Figure S19** **– Heatmaps showing differential nuclear DNA-encoded gene expression between the LE and HE alpha cells subgroups (Datasets II and III).** Heatmap of significant differentially expressed nDNA OXPHOS genes (left) and GCG gene (right) per cluster per dataset (purple- low expression, yellow- high expression), High and low mtDNA gene expression sign as HE and LE, respectively. Unique mapping results of (A) Datasets II (B) Dataset III. Non-unique mapping results of (C) Datasets II (D) Dataset III.


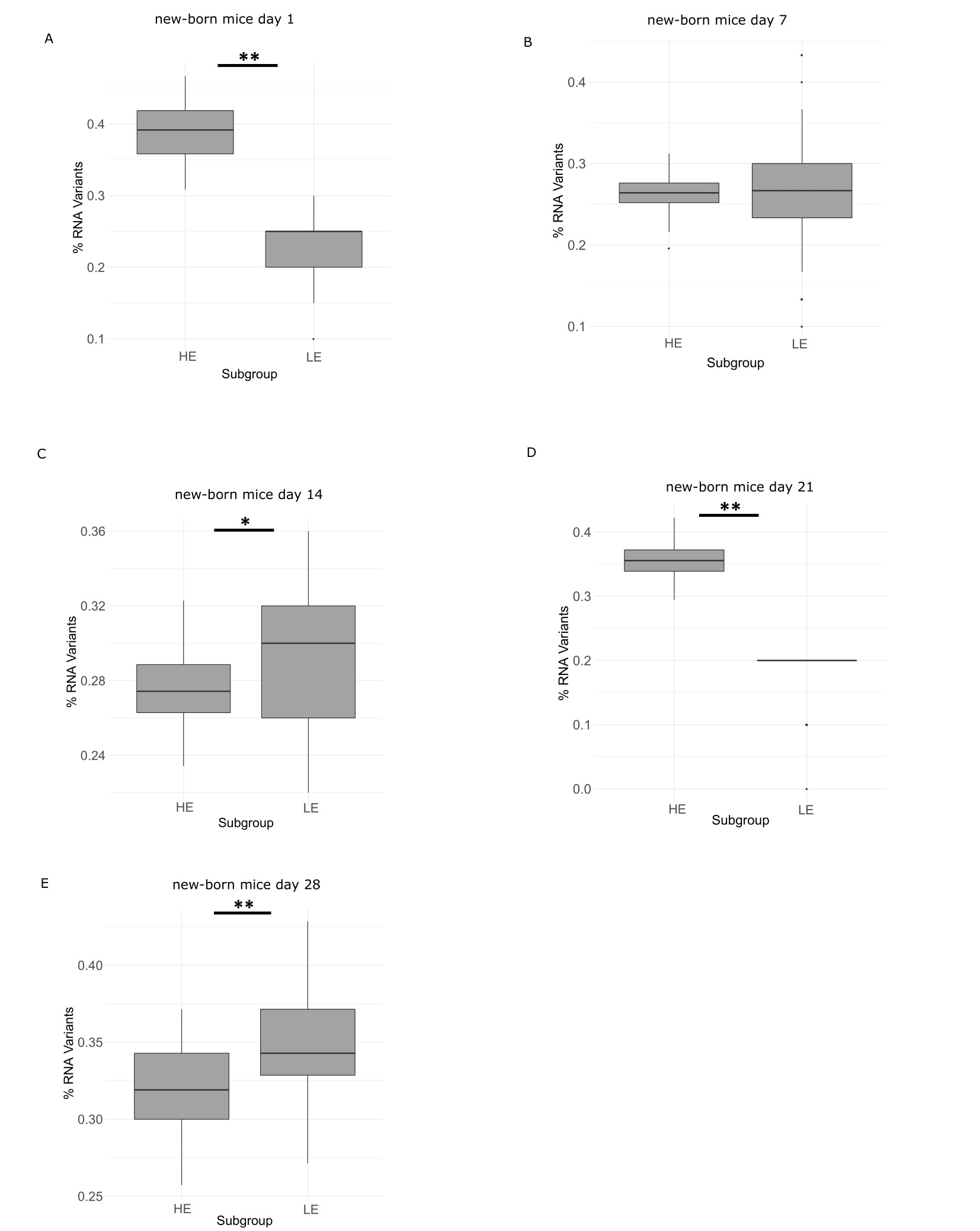


**Figure S20** **- mt-RNA mutational pattern analysis in mouse Dataset III - unique mapping.** (A-E) Box plots representing the percentage of mtRNA mutation variants in each of the cell groups per sampling iteration (see Online Methods) of mouse Dataset III (Significant values: *-p<0.005, **-p<1x10^-10^).


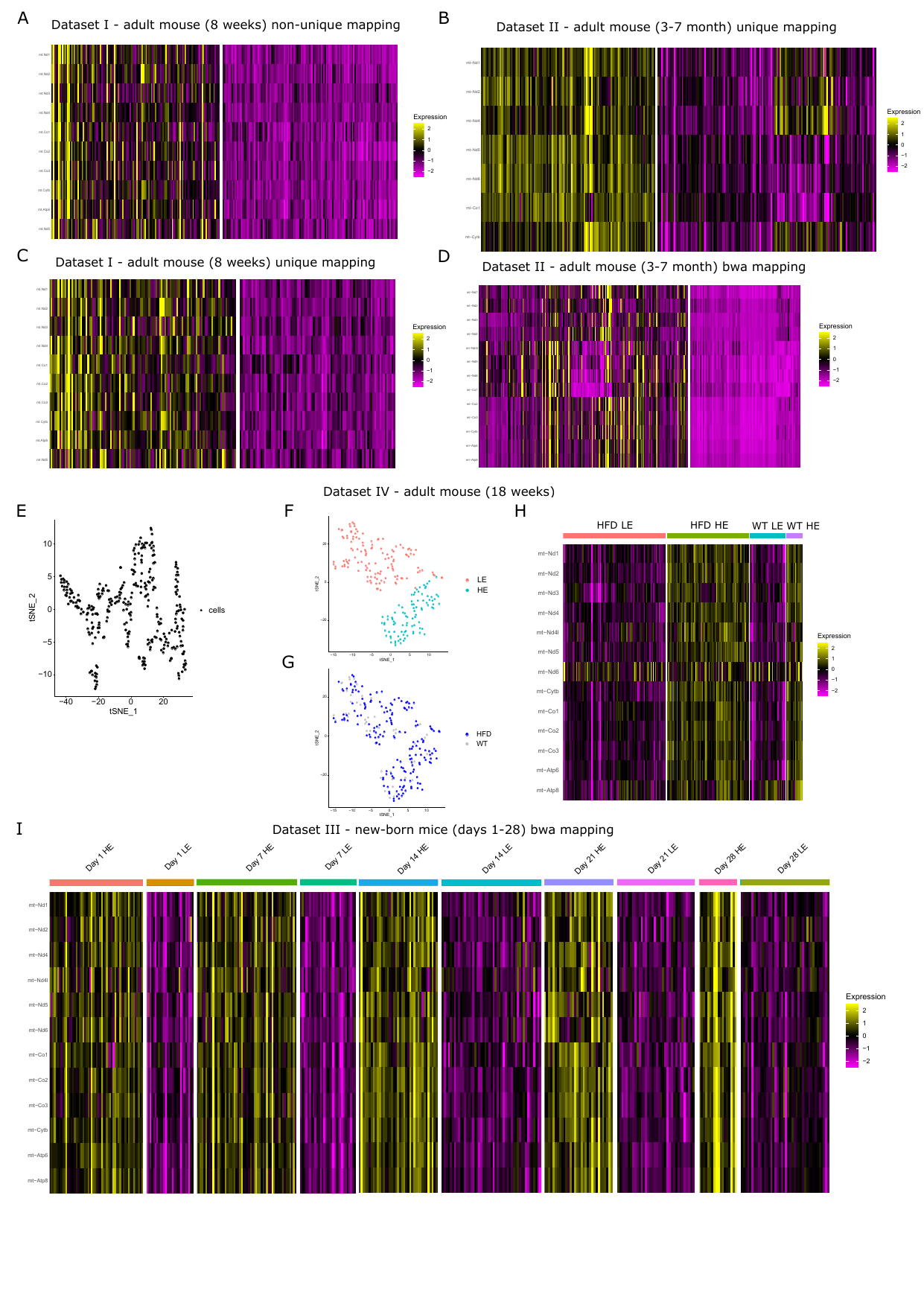


**Figure S21** – **mtDNA gene expression analysis of adult mice do not display consistent subgroups of beta cells, while new-born mice displayed two subgroups of beta cells according to mtDNA gene expression.** Heatmaps of adult and postnatal mice (purple- low expression, yellow- high expression) showing the mtDNA gene expression per mapping method (e.g. bwa mapping – mapped solely against the mtDNA, unique mapping and non-unique mapping –against the entire genome). (A) Eight weeks old mice – mouse Dataset I (non-unique mapping), (B) Adult 3-7 month old mice – mouse Dataset II (unique mapping), (C) Adult 8 weeks old mice – mouse Dataset I (unique mapping), (D-E) Adult 3-7 month old mice – mouse Dataset II (bwa mapping), (F-H) Adult 18 weeks-old mice – mouse Dataset IV (n=217 high quality cells with an average of ~4300 informative genes per cell): (F) tSNE display of beta cells subgroups, i.e. with either low (LE) or high mtDNA gene expression (HE), (G) tSNE showing the distribution of beta cells subgroups for high fat diet (HFD) and control diet mice. Blue: HFD, grey: Control. (H) Heatmap showing the mtDNA gene expression subgroups for HFD and control mice. (I) Heatmap of postnatal mice – Dataset III (bwa mapping).


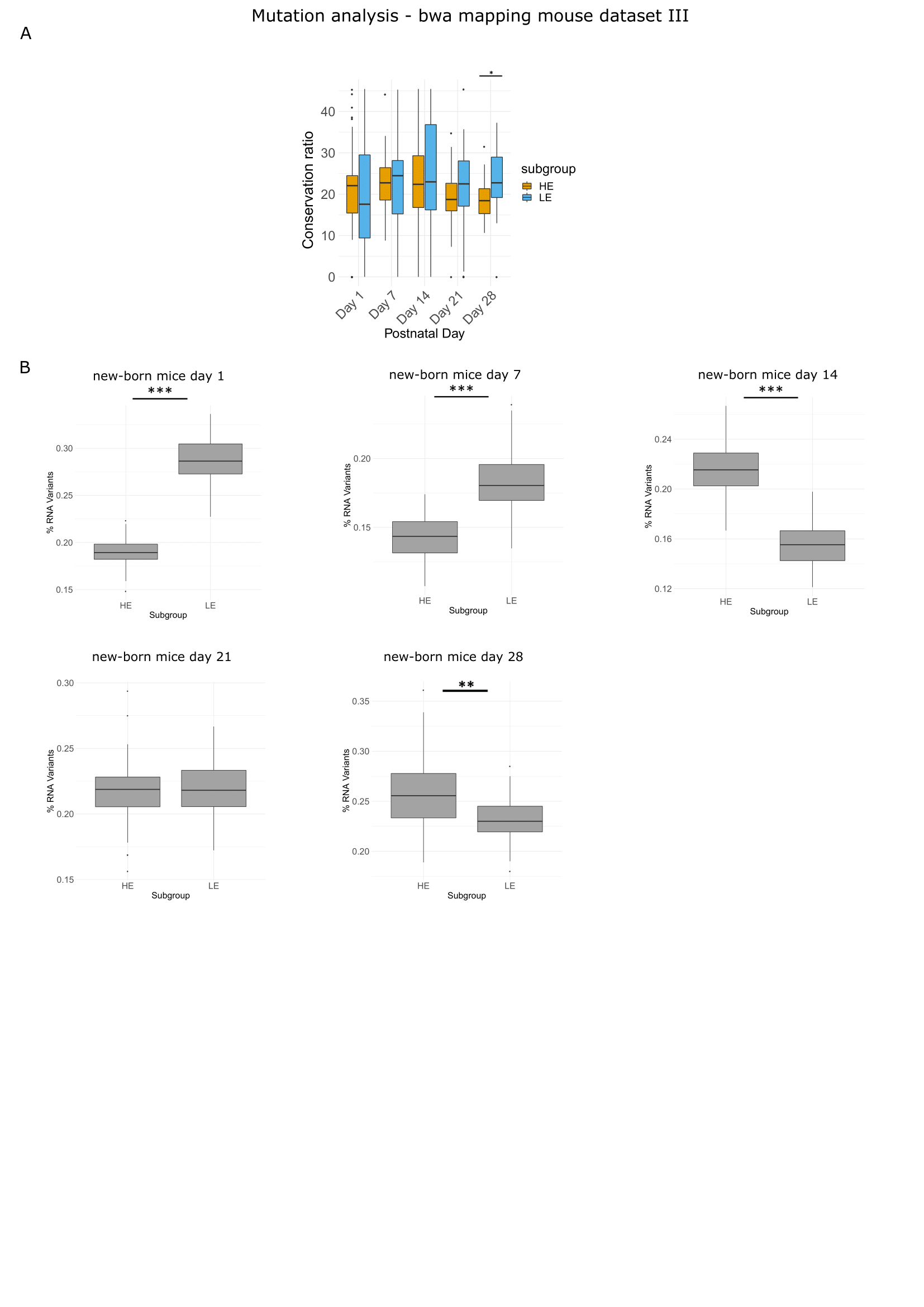


**Figure S22 – mt-RNA mutational pattern analysis- bwa mapping solely against the mtDNA genome of mouse Dataset III.** (A) Box plot of the conservation ratio distribution in mutations within the LE and HE cellular subgroups. (B-F) Box plot of percentage of mtRNA mutation variants in each of the groups per 10^3^ sampling iteration (Significant values: *- p<0.05, **-p<1x10^-7^, ***-p<1x10^-26^).
